# Pattern of antibiotic use and bacterial co-infection in hospitalized Covid-19 patients

**DOI:** 10.1186/s43168-023-00195-5

**Published:** 2023-03-31

**Authors:** Salma Said Zaki, Gamal El Sawaf, Asmaa AbelHameed Ahmed, Ayman Ibrahim Baess, Bassem Nashaat Beshey, Amel ELSheredy

**Affiliations:** 1 Microbiology Department, Medical Research Institute, Alexandria University, Alexandria, Egypt; 2 Biomedical Informatics and Medical Statistics Department, Medical Research Institute, Alexandria University, Alexandria, Egypt; 3 Chest Diseases Department, Faculty of Medicine, Alexandria University, Alexandria, Egypt; 4 Critical Care Medicine Department, Alexandria Faculty of Medicine, Alexandria, Egypt

**Keywords:** Covid-19, Antibiotic, Cultures, ICU

## Abstract

**Background:**

There is evidence that bacterial co-infection in respiratory viruses leads to morbidity and mortality. Patients with decreased immunity are prone to bacterial co-infection. A lack of judicious use of antibiotics leads to the spread of multi-drug resistant bacteria (MDR) that have a long-term negative impact. In this study, we attempted to observe the pattern of antibacterial use and its impact on secondary bacterial infection.

**Methods:**

An observational study was conducted at Alexandria Main University Hospital (AMUH) (Alexandria University) from June 2021- February 2022. Study participants were admitted to the Intensive Care Unit (ICU) with confirmed Covid-19 (by Polymerase Chain Reaction (PCR) and Computed tomography (CT) scan). The following data was collected (Demographic, clinical, and laboratory data).In this study, the Pattern of antibiotic use as well as the occurrence of secondary bacterial infections were reported.

**Results:**

Among 121 patients included in the present study, all received antibiotics empirically. Upon admission (19.8%) showed urinary tract infection, (11.5%) had bloodstream infection, and (57.7%) had respiratory tract infection. After 10 days secondary bacterial infection occurred in 38 patients (61.2%) with (24.1%) Urinary tract infection (UTI), (12.9%) Bloodstream infection (BSI), and (72.2%) respiratory tract infection. The respiratory sample size was (45) patients due to Infection Control (IC) restrictions on the aerosol-producing procedure.

**Conclusion:**

Upon admission, all patients received broad-spectrum antibiotics while the incidence of bacterial co-infection was low.

## Introduction

Bacterial coinfections are a major cause of morbidity and mortality among patients with respiratory tract infections of viral origin as the Influenza virus [1]. There is a great challenge to distinguish between a viral and bacterial infection that leads to unnecessary use of antibiotics [2].

During the SARS-CoV-2 pandemic, There has been a multipronged increase in antimicrobial prescriptions in patients who present with severe COVID-19, despite a low prevalence of bacterial coinfections on admission (3.5%–8%) [3]. Several factors contribute to this problem, including the difficulty of obtaining respiratory samples, the breakdown of surveillance and antimicrobial stewardship programs, and the lack of evidence-based antiviral therapies [4].

Broad-spectrum antibiotics are prescribed empirically upon ICU admission due to the fear of bacterial co-infection of the respiratory tract as a consequence of ICU stay. COVID-19 was treated with antibiotics without obtaining culture-based results, which may have resulted in the development of MDR and the return to pre-antibiotic times. The substantial increase in the use of broad-spectrum antibiotics from the "watch" or "reserve" categories will render them ineffective, resulting in highly drug-resistant clusters that could create a difficult situation for clinicians. This threatens antimicrobial stewardship [5] For example, an increase in the use of azithromycin, a broad-spectrum macrolide antibiotic, has been documented in many African countries. So the current study aimed to show the pattern of antibiotic use and bacterial co-infection in hospitalized covid-19 patients and susceptibility patterns of the causing bacterial isolate [6].

### Materials and methods

The observational study took place during the period from (June 2021-February 2022) During the course of the study, data was collected from (121) patients who were admitted to the Alexandria Main University Hospital (AMUH) ICU following confirmation of a COVID infection according to the criteria of the Egyptian Ministry of Health by PCR and (patient risk factors, level of oxygenation, degree of CT involvement,worsening of general conditions). In this study, data were collected to determine the antibiotics used, the occurrence of bacterial co-infections, the causative organisms, and their antibiotic susceptibility pattern.

### Data collection

#### Data were collected from patient files that included:

- Demographic data (Age, sex)
- Comorbidities (COPD, asthma, heart failure, Atrial fibrillation, Liver cirrhosis, Diabetes mellitus, Chronic kidney disease, End-stage renal disease on dialysis, Coronary artery disease, Hypertension, Obesity),
- Medications prescribed (steroid, antimicrobials, anti-Interleukin 6), type of antibiotics
- Laboratory data (Hemoglobin, WBC, ….) for all patients were collected on admission and after 10 days
- Clinical outcome. (need for intubation, need for hemodialysis, death)

### Sample collection

Samples (blood, urine, respiratory sample (Bronchoalveolar Lavage BAL/sputum)) were taken upon admission and after 10 days of ICU stay.

### Microbial detection

- Samples were cultured on MacConkey agar and blood agar and incubated at 37oc for 24–48 h
- Plates were examined for bacterial growth
- Identification of grown colonies by microscopical examination and standard biochemical tests
- Disc diffusion method was used to determine antibiotic susceptibility by measuring the inhibition zone according to CLSI guidelines (Clinical and Laboratory Standards Institute).

### Statistical analysis

Data were analyzed to determine the different patterns of antibiotic use and their effect on the occurrence of secondary bacterial infection in patients using the Statistical Package for Social Sciences (SPSS ver.25 Chicago, IL, USA). Paired t-test was used for the comparison of quantitative variables at admission and follow-up. McNemar test was used to test differences in proportions at admission and follow-up. Kaplan Meier survival analysis was used for survival analysis.

## Results

It is noteworthy that the participants in this study were primarily male (52.8%) with a mean age of (64.7 ± 14.7) years. It was found that 48.7% of patients had died by day 10 of their ICU stay. Only one patient received the covid vaccine before admission (Table 1).

**Table 1:** Demographic data and main characteristics of the 121 participants involved in the study

Main Characteristic	Number (%)
**Age**	
Mean ± SD	64.7 ± 14.7
(Min–Max)	(18–92)years
**Sex**	
Female	57(47.2%)
Male	64(52.8%)
**Outcome (On day 10)**	
Death	59(48.7%)
Alive	62(51.3%)
**Mechanical ventilation**	
Yes	59(48.7%)
No	62(51.3%)
**COVID vaccine**	
Yes	1(0.08%)
No	120(99.12%)

**Fig. 1:**
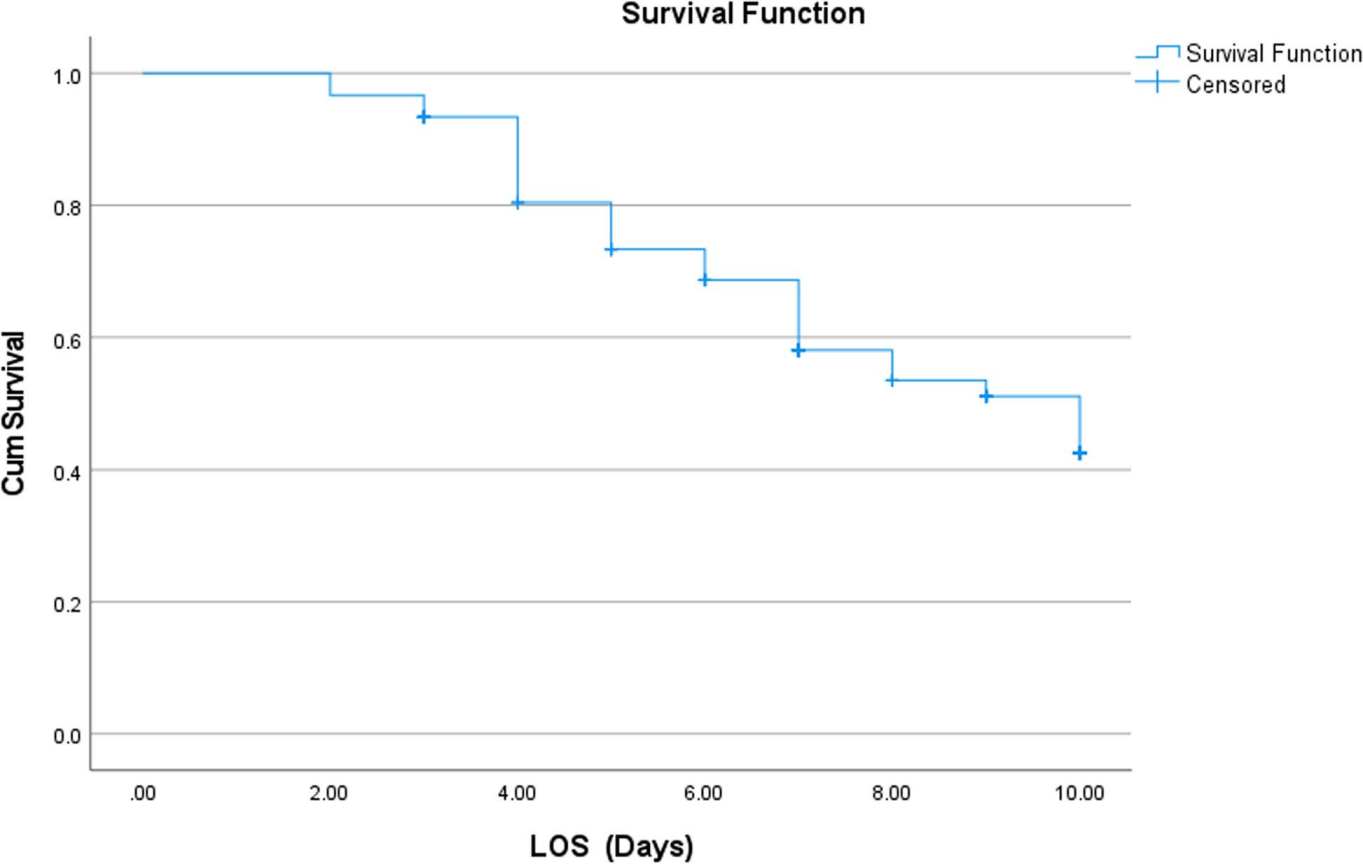
Kaplan–Meier survival curve for the length of stay till death for 121

According to the Kaplan–Meier survival curve, 62 patients survived (51.3%), while 59 patients died (48.7%) (Fig. 1).

**Fig. 2:**
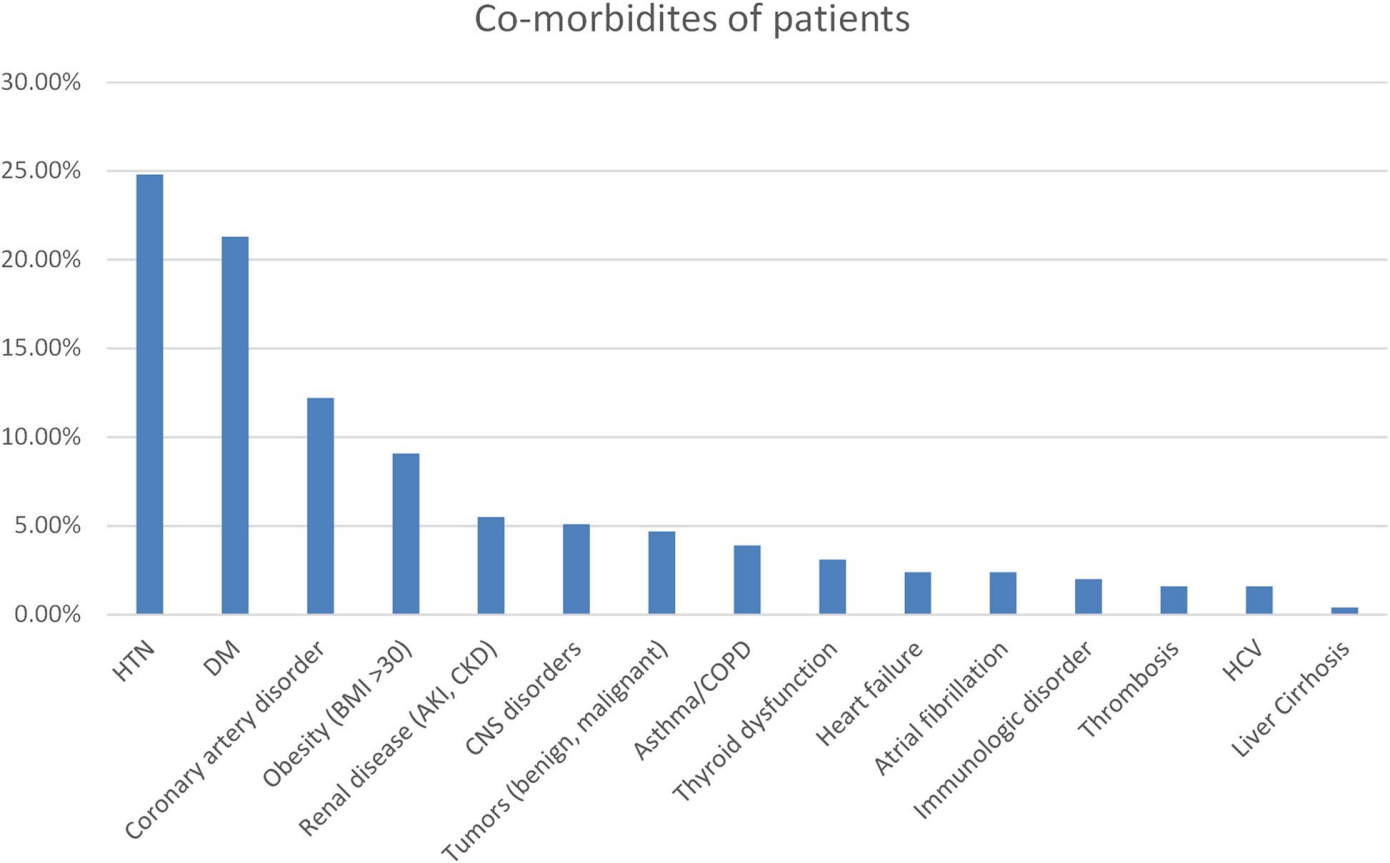
Comorbidities of the 121 participants involved in the study

In most cases, patients included in the study had multiple combined comorbidities, mainly diabetes mellitus (DM) and hypertension (HTN). Also, obesity found in 19% of the participants (Body mass index = 30). Among the comorbidities found, hypertension (HTN) was the most prevalent, followed by diabetes mellitus (DM), Coronary artery disease, obesity, renal disease, and a central nervous system (CNS) disorder (Fig. 2).

Bacterial coinfections also cause hematological and biochemical imbalances, worsening the general clinical condition (Table 2). The mean of IL-6 in patients was (291.78) the mean D- dimer was (721.68), mean Ferritin was (397.3). The regression model predicting infection was statistically significant (X^2^ = 34.2, *p* < 0.001) Significant predictors were gender (Odds Ratio = 3.23, *p* value = 0.042), while other predictors were not statistically significant (Table 3).

**Table 2:** Laboratory results of patients at admission and at day 10

	**Upon admission**	**On day 10**	***p***-value
**Hemoglobin**			
Mean ± SD	11.9±2.2	11.3±2.1	< 0.001*
**Platelets**			
Mean ± SD	239.7±107	237.9±119	0.86
**Total leucocyte count**			
Mean ± SD	12.2±6.8	13.9±7.6	0.014*
**Lymphocytes**			
Mean ± SD	1.6±1.4	1.4±1.4	0.206

**Table 3:** The regression model predicting infection

	B	S.E	*P*	OR	95% C.I. for OR	
					Lower	Upper
**Age**	-.003	.013	.845	.997	.972	1.023
**sex**	1.130	.556	.042*	3.23	1.09	8.61
**DM**	-.561	.583	.336	.570	.182	1.789
**HTN**	-.027	.555	.961	.973	.328	2.889
**Renal**	-.845	.791	.286	.430	.091	2.025
**Heart failure**	-.827	1.178	.483	.437	.044	4.399
**Thyroid dysfunction**	-.838	.862	.331	.433	.080	2.345
**Asthma/COPD**	-.827	.878	.347	.437	.078	2.447
**Hb1**	.076	.082	.355	1.079	.919	1.266
**WBCS1**	.020	.039	.598	1.021	.946	1.101

*OR* Odds ratio, *SE* Standard error

^*^statistically significant

Among the empirical antibiotics prescribed with a wide range, Combined therapy was used that contained either Levofloxacin or Meropenem (mostly prescribed antibiotics) according to the IDSA (The Infectious Diseases Society of America) guidelines for HAP(Hospital Associated Pneumonia)/VAP(Ventilator Associated Pneumonia) (Table 4).

**Table 4:** Antibiotics prescribed to patients during ICU stay

Antibiotics	Responses		Cumulative percentage of Cases
	**N**	**Percent**	
Levofloxacin	70	21.10%	59.30%
Meropenem	59	17.80%	50.00%
Linezolid	55	16.60%	46.60%
Cefepime	42	12.70%	35.60%
Clarithromycin	23	6.90%	19.50%
Ceftazidime	16	4.80%	13.60%
Piperacillin/Tazobactam	14	4.20%	11.90%
Ceftriaxone	14	4.20%	11.90%
Amikacin	6	1.80%	5.10%
Tigecycline	5	1.50%	4.20%
Moxifloxacin	5	1.50%	4.20%
Imipenem/Cilastatin	4	1.20%	3.40%
Teicoplanin	4	1.20%	3.40%
Ciprofloxacin	3	0.90%	2.50%
Colistin	3	0.90%	2.50%
Doxycycline	3	0.90%	2.50%
Azithromycin	3	0.90%	2.50%
Clindamycin	2	0.60%	1.70%
Vancomycin	1	0.30%	0.80%
Total	332	100.0%	281.4%

At the time of admission, 63 patients had bacterial co-infection(52%) (Table 5). Urinary tract infection (UTI) was reported by 19.8% of the patients, with *E. coli* being the most commonly isolated organism. Blood Stream Infection (BSI) was detected in 11.5% of patients whose most common isolate was *Coagulase-negative staphylococcus.* While respiratory tract infection was observed in (57.7%) of patients carrying the most common isolate *Klebsiella spp* (Table 6).

**Table 5:** Results of cultures of (blood, respiratory sample, urine) of patients upon admission to the ICU and on day 10 of ICU stay

Culture type	Upon admission (64)	On day 10 (2ry bacterial infection) (39)
**Urine**	19.8% positive (24/121)	24.1% positive (15/62)
**Blood**	11.5% positive (14/121)	12.9% positive (8/62)
**Respiratory sample**	57.77% positive (26/45)	72.2% positive (16/22)

**Table 6:** Bacteria isolated from patients at admission and at day 10

Bacteria isolated	Urine Upon admission (24)	Urine on day 10 (*n* = 15)	Blood On admission (*n* = 14)	Blood On day 10 (8)	Respiratory On admission (*n* = 26)	Respiratory On day 10 (*n* = 16)
*Coagulase-negative Staphylococcus*	1 (4.16%)	1 (6.67%)	4 (28.57%)	1 (12.5%)	1(3.84%)	0%
*Enterococcus spp*	2 (8.32%)	1 (6.67%)	–-	–-	1 (3.84%)	0
*E-Coli*	12 (50%)	7 (46.67%)	1 (7.14%)	0%	5 (19.23%)	2 (12.5%)
*Pseudomonas spp*	4 (16.66%)	2 (13.3%)	2 (14.28%)	2 (25%)	3 (11.5%)	3 (18.75%)
*Klebsiella spp*	5 (20.8%)	4 (26.67%)	1 (7.14%)	1 (12.5%)	9 (34.61%)	5 (31.25%)
*MRSA*	–-	–-	3 (21.42%)	3 (37.5%)	–-	–-
*Streptococcus Pneumonia*	–-	–-	1 (7.14%)	0%	2 (7.69%)	1 (6.25%)
*Acinetobacter*	–-	–-	2 (14.28%)	1 (12.5%)	3 (11.5%)	3 (18.75%)
*Proteus*	–-	–-	–-	–-	1 (3.84%)	1 (6.25%)
*Stenotrophomonas maltophilia*	–-	–-	–-	–-	0%	1 (6.25%)
*Staphylococcus Aureus*	–-	–-	–-	–-	1 (3.84%)	0

Thirty-eight patients developed secondary bacterial infections after ten days. This was identified by new onset of fever, worsening of the patient’s general condition, increase in C-reactive protein (CRP) besides WBC elevation and ragiologically by new onset of denove infilterates or consolidations. After 10 days secondary bacterial infection occurred in 38 patients (61.2%) with (24.1%) of them developing Urinary Tract Infection (UTI) caused by the most common organism *E.Coli*. The most common isolated organism in bloodstream infections was *MRSA* with a percentage (of 12.9%). While respiratory tract infection was observed in (72.2%) of patients carrying the most common isolate *Klebsiella spp *(Table 6).

## Discussion

The present study was carried out to show the pattern of antibiotic use and bacterial co-infection in hospitalized Covid-19 patients. This study was carried out on 121 patients admitted to the ICU Alexandria University Main Hospital with signs and symptoms of COVID-19 and confirmed by PCR and chest CT. Previous studies as in Petrilli, et al., 2020 [7] and Killerby, et al., 2020 [8] showed that increasing the severity of the disease occurs in old age in agreement with our study the mean age of the patients included in the study was (64.7 ± 14.7) years.

In our study males represented (52.8%). There is a strong association between older age, male sex with hospital admission, and liability of critical illness among all patients with covid-19 [9].

Mechanical ventilation increases the risk of mortality in patients. During ICU stay (48.7%) of our patients needed mechanical ventilation. In agreement with Silva, et al., 2021 [10] and Petrilli, et al., 2020 [7] where (53.8%) and (65.35%) of their patients required mechanical ventilation respectively.

There are proposed theories that patients with cardiometabolic diseases may have higher rates of bacterial coinfection during viral infection due to an underlying immune dysfunction. In the present study, Hypertension was the most prevalent comorbidity among our patients (24.8%) agreed with Killerby, et al., 2020 [8] and Garg, et al., 2020 [9].

The second most common comorbidity among our patients was Diabetes. Diabetes mellitus is known to decrease effective T-cell and neutrophil response [11]. In our study diabetic patients with diabetes represented (21.3%).

Obesity is another comorbidity among our patients that is associated with a high risk of death in patients. Obesity is known to be a pro-inflammatory condition and induces diabetes and oxidative stress that cause cardiovascular dysfunction and impair the immune response to viral infection. A Percentage of (19%) of our patients had BMI = 30. In agreement with [12] and studies whereby Soares, et al., 2020 [13] (18.9%) were obese.

Bacterial coinfections also cause hematological and biochemical imbalances, worsening the general clinical condition [14]. In the current study, the mean Hemoglobin upon admission was 11.9 ± 2.2 and after 10 days 11.3 ± 2.1, there was no significant decrease in Hemoglobin. This was against Moriyasu Anai et al. 2021, [15] where there was a significant decrease in hemoglobin in severe cases. Decreased Hb level after pneumonia diagnosis in COVID-19 patients might lead to severe respiratory failure and the need for mechanical ventilation [15].

Leucopenia is characteristic of early COVID with bacterial co-infection. With increasing disease severity, the leukocyte count increases significantly. In this study, the increase in total leucocyte count was statistically significant. This agreed with Kazemi, et al., 2021 [16].

Mean D- dimer of our patients upon admission was high (721.68) which agreed with Petrilli, et al., 2020 [7] where the mean D-dimer was (528). D- dimer which is an indicator of blood clotting liability. Elevated D-dimer upon admission was related to a poor prognosis of COVID-19 [17]. The mean IL-6 in our patients was (291.78). Elevated levels are recorded in severe-to-critical COVID-19 patients [18].

Many comorbidities increase the severity of COVID-19 disease which may lead to death. Bacterial infection also may increase the risk of death. The death occurred in (48.7%) of our patients before day 10 of the study while (51.3%) survived. In Gupta, et al., 2020 [19] and Gasperini, et al., 2021 [20] death occurred in (35.4%) and (32.9%) of their patients respectively that agreed with our findings.

During an ICU stay, a combination of antibiotics was prescribed as an empirical treatment to minimize the risk of secondary bacterial infection, specifically when mechanical ventilation is used. As part of the present study, all patients received empirical antibiotic therapy. levofloxacin was prescribed to 70% of the enrolled subjects. The most common combination used in our study was (Levofloxacin and Linezolid).In Goncalves Mendes et al. 2020, only 67% of patients received empirical therapy (Cefepime (45%), Ceftriaxone (54%), Vancomycin (48%), and Azithromycin (47%)) were used [21].

Cultures were withdrawn (urine, blood, respiratory sample sputum/ BAL) from patients upon admission and on the tenth day of admission. Upon admission, positive urine cultures were (19.8%) and on day 10 (24.1%). In Li, et al., 2020 [17] (7.8%) of patients had Urinary tract infection as a secondary infection. The high prevalence of Urinary tract infections may be explained by underlying Diabetes and the use of urethral catheters [22].

In our study, the most prevalent bacteria isolated from urine were *E Coli* (50%), *Klebsiella* (20.8%), and *Pseudomonas* (16.66%) upon admission respectively. In agreement with previous studies as in Karaba, et al., 2021 [23]. in Li, et al., 2020 [17] the main isolated pathogen in Urinary tract infection was *E-Coli*.

Blood cultures were positive in 11.5% of patients upon admission and in 12.9% of patients on day ten. In agreement with Miyagami, et al. 2021 [24] (17.0%) of patients showed positive blood cultures. Li, et al., 2020 [17] and (34.3%) had bloodstream infection as a secondary bacterial infection, In our study, the most common bacteria isolated from blood culture were *Coagulase Negative Staphylococcus* (28.57%), *MRSA* (21.42%), *Pseudomonas* (14.28%), *Acinetobacter* (14.28%) upon admission respectively. *Coagulase Negative Staphylococcus* might be a contaminant or a true pathogen. A higher percentage of isolation of *Coagulase Negative Staphylococcus* from blood cultures was by Sepulveda, et al., 2020 [25] and Martinez-Guerra, et al., 2021 [26]. In Miyagami, et al. 2021 [24] It was determined that (E. coli, Klebsiella Pneumonia, and Staphylococcus aureus) were the most prevalent bacteria isolated from blood cultures.

Obtaining acceptable respiratory samples for bacterial identification and other microbiological studies is limited due to concerns about aerosol-producing procedures.

Among our patients, only 45 respiratory samples were collected. A majority of patients (57.77%) displayed positive cultures upon admission and more than seventy percent (72.2%) had a positive respiratory sample by day ten. In Li, et al., 2020 [17] (86.3%) of patients had respiratory tract infections. In Langford, et al., 2021 [27] (38.22%) of patients had a respiratory infection. The respiratory cultures found in our study were 34.61% for *Klebsiella* 19.23% for *E-coli*, 11.5% for *Acinetobacter*,and 11.5% for *Pseudomonas*.

According to our results, 31 percent of all the bacteria isolated during this study (32/103) were multidrug-resistant (MDR) bacteria, which were divided as follows: nine *E. coli*, seven *Klebsiella*, six *Pseudomonas*, Five *Acinetobacter*, three *Coagulase Negative Staphylococcus,* and one *MRSA*. In agreement with Polly, et al., 2022 [28] the percentage of MDR culture results was (29.7%). While in Gasperini, et al., 2021 [20] (55.4%) of culture results were MDR. We found four cultures that were PAN-resistant in our study. A PAN *Pseudomonas* isolate was obtained from a respiratory sample, a PAN *Klebsiella pneumonia* isolates was obtained from a respiratory sample, a PAN *E.coli* isolate was obtained from a respiratory sample and a PAN *Acinetobacter* isolate was obtained from a blood sample.

We acknowledge several limitations in this study. Data about antibiotics the patients received before ICU admission was lacking. Also, The sample size wasn’t big enough for more significant results. More follow-up for patients was needed. Additionally, due to concerns about aerosol generation, respiratory samples (BAL/ sputum) weren’t withdrawn from all patients.

## Conclusion

There is a high prevalence of empiric antibiotic treatment in patients admitted with severe COVID-19 infection. Urinary tract infections and Bloodstream infections were the most common hospital-acquired infection due to *E.Coli* and *Methicillin-resistant Staphylococcus Aureus (MRSA)* organisms. The impact of COVID-19 pandemic on antimicrobial resistance needs further investigations.

## Data Availability

All data generated or analyzed during this study are included in this published article.
